# A SELEX-Screened Aptamer of Human Hepatitis B Virus RNA Encapsidation Signal Suppresses Viral Replication

**DOI:** 10.1371/journal.pone.0027862

**Published:** 2011-11-18

**Authors:** Hui Feng, Jürgen Beck, Michael Nassal, Kang-hong Hu

**Affiliations:** 1 State Key Laboratory of Virology, Wuhan Institute of Virology, Chinese Academy of Sciences, Wuhan, China; 2 University Hospital Freiburg, Internal Medicine II/Molecular Biology, Freiburg, Germany; Yonsei University, Republic of Korea

## Abstract

**Background:**

The specific interaction between hepatitis B virus (HBV) polymerase (P protein) and the ε RNA stem-loop on pregenomic (pg) RNA is crucial for viral replication. It triggers both pgRNA packaging and reverse transcription and thus represents an attractive antiviral target. RNA decoys mimicking ε in P protein binding but not supporting replication might represent novel HBV inhibitors. However, because generation of recombinant enzymatically active HBV polymerase is notoriously difficult, such decoys have as yet not been identified.

**Methodology/Principal Findings:**

Here we used a SELEX approach, based on a new *in vitro* reconstitution system exploiting a recombinant truncated HBV P protein (miniP), to identify potential ε decoys in two large ε RNA pools with randomized upper stem. Selection of strongly P protein binding RNAs correlated with an unexpected strong enrichment of A residues. Two aptamers, S6 and S9, displayed particularly high affinity and specificity for miniP *in vitro*, yet did not support viral replication when part of a complete HBV genome. Introducing S9 RNA into transiently HBV producing HepG2 cells strongly suppressed pgRNA packaging and DNA synthesis, indicating the S9 RNA can indeed act as an ε decoy that competitively inhibits P protein binding to the authentic ε signal on pgRNA.

**Conclusions/Significance:**

This study demonstrates the first successful identification of human HBV ε aptamers by an *in vitro* SELEX approach. Effective suppression of HBV replication by the S9 aptamer provides proof-of-principle for the ability of ε decoy RNAs to interfere with viral P-ε complex formation and suggests that S9-like RNAs may further be developed into useful therapeutics against chronic hepatitis B.

## Introduction

Hepatitis B virus (HBV), the prototypic member of the *Hepadnaviridae*, is the causative agent of B-type hepatitis [1]. The enormous number of chronic HBV carriers and their greatly increased risk to develop severe liver disease, including liver cirrhosis and hepatocellular carcinoma (HCC) [2], make chronic HBV infection a major worldwide public health problem [3], [4]. Currently approved therapies suffer from low response rates, severe adverse effects and a high rate of drug resistance [4]–[6]. Hence new targets for antiviral therapy need to be defined so as to provide an armory of different strategies that, in combination, may lead to life-long suppression or even elimination of virus replication.

A unique characteristic of HBV replication is the protein-primed reverse transcription of an RNA intermediate, the pregenomic RNA (pgRNA), which takes place within viral capsids (core particles) [7]–[9]. Assembly of such replication-competent capsids requires the highly selective co-packaging of pgRNA with the viral polymerase, a reverse transcriptase (RT) called P protein [10]–[13]. Critical to this packaging process is the specific recognition and formation of a ribonucleoprotein (RNP) complex between P protein and an RNA stem-loop, ε, close the 5′-end of the pgRNA [11], [14], [15]. Beyond packaging, formation of the P-ε complex is necessary for the initiation of reverse transcription via protein priming [16], [17]. Inhibiting this crucial interaction should block viral replication at both the pgRNA packaging and reverse transcription levels, and hence represents a highly attractive novel strategy for therapeutic intervention.

Aptamers are the high affinity ligands derived from libraries of randomized molecules through ″SELEX″ (Systematic Evolution of Ligands by Exponential Enrichment), a high-flux screening technique involving repeated rounds of partitioning and amplification [18], [19]. As a promising class of compounds with high affinity, specificity and stability, aptamers have been selected for a wide range of targets, from small organic molecules to complex proteins or even intact cells [20]–[23]. Furthermore, these advantages expand the possible applications of aptamers to include their use as therapeutics and diagnostics [24]–[26]. A first aptamer-based drug has already been approved in the treatment of ocular vascular disease [27].

Previously, the feasibility of identifying aptamers specifically binding a hepadnaviral P protein by *in vitro* selection has been demonstrated for the related duck HBV (DHBV) after recombinant DHBV P protein had successfully been reconstituted into priming-active RNPs [17], [28]; the *in vivo* effects of aptamer sequences replacing the authentic ε-sequence in the DHBV genome have recently been reported [29], [30]. For human HBV, however, *in vitro* SELEX-based screening for such aptamers was not possible until very recently, when Hu and coworkers succeeded in reconstituting RNP formation with HBV P protein *in vitro* [31]; the RNPs appeared as slowly migrating material in RNA electrophoretic mobility shift assays (EMSAs). Even though the RNPs lack enzymatic activity, a modification of this reconstitution system enabled us to set up an *in vitro* SELEX procedure by which we successfully isolated high-affinity RNA aptamers against recombinant truncated HBV P protein (miniP) from two large RNA pools. In one pool (termed AS), the upper ε stem was completely randomized, in the other (termed S), the naturally conserved apical loop sequence was maintained. Among various strongly binding aptamers, the one with the highest affinity and specificity for P protein, S9, inhibited HBV replication strongly in transiently cotransfected HepG2 cells, and still substantially in the stably HBV producing HepG2.2.15 line. As shown below, this inhibition occurs most likely by competition of the aptamer with the authentic ε signal on pgRNA. The *in vitro* SELEX-based aptamer selection thus represents a powerful strategy to identify decoys that might become therapeutically applicable to reduce viral loads in chronic HBV infection.

## Results

### Expression, purification and functional characterization of the miniP protein

**Figure 1:**
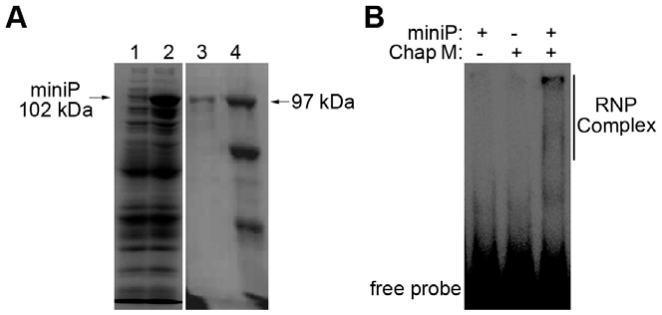
Purification and functional characterization of the miniP protein. (A) Expression and purification of the miniP protein. MiniP was expressed and purified as His-tagged MBP fusion protein in E. coli, and detected by SDS-polyacrylamide gel electrophoresis followed by Coomassie blue staining. Lane 1 and 2: BL21- CodonPlus(DE3) cells without and with IPTG induction; Lane 3: purified miniP protein; Lane 4: marker proteins with their molecular masses indicated in kDa. (B) *In vitro* binding capacity of the miniP as detected by EMSA. Chap M refers to a mixture of chaperones as described in the text. ^32^P-labeled free ε RNA (probe) and miniP-ε complexes (RNP) are indicated.

In order to acquire sufficient amounts of soluble, ε binding-active HBV P protein, we employed an MBP-fused and His-tagged miniP protein in which the dispensable spacer region (aa 200-291) and the C terminal 231 aa including the RNase H domain were deleted. Analogous DHBV miniP constructs display authentic, ε-dependent priming activity [32]. The HBV miniP was expressed in *E. coli* strain BL21-CodonPlus(DE3) and purified using immobilized metal affinity chromatography (IMAC) performed as previously described for the DHBV P protein (Fig. 1A) [33], [34].

*In vitro* priming activity of near full-length DHBV P protein requires the chaperones Hsp70 and Hsp40 plus energy, and is further stimulated by Hsp90 and its co-chaperones Hop and possibly p23 [35], [36]. This chaperone dependence is lost in DHBV miniP [33], [36] yet *in vitro* ε binding activity of HBV miniP proteins similar to the one used here reportedly is dependent on, or at least promoted to detectable levels by, the combined Hsp70/Hsp90 systems [31], [37]. We therefore used a similar reconstitution assay including purified Hsp90, Hsc70, Hop, Hdj-1 and p23 to test whether our miniP protein is functional in ε binding. As shown in Fig. 1B, the reaction setup with both miniP protein plus chaperones demonstrated upward shifted signals similar to those previously reported [31], which were not observed in the absence of either miniP or chaperones, consistent with a specific miniP–ε RNA interaction. The absence of His-tags from all chaperones used in the current study should then allow to specifally capture the His-tagged miniP and bound RNAs by IMAC, as required for the subsequent SELEX experiments.

### Selection of HBV miniP binding aptamers from upper stem-randomized RNA pools

**Figure 2:**
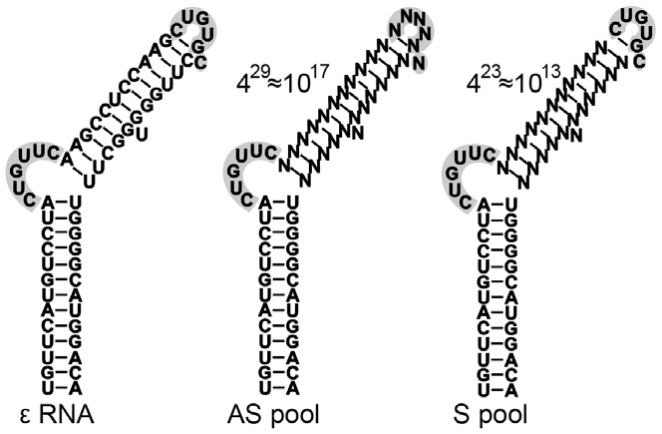
Randomized regions in the starting RNA pools. The secondary structure of wt HBV ε, with its lower stem, upper stem, bulge and apical loop (both highlighted by grey shading) is shown on the left. The bulge contains the template for replication initiation. In the AS pool, the entire 29 nt upper stem was randomized (indicated by Ns). In the S pool, the sequence forming the apical loop was preserved. The nominal number of possible individuals in each pool is also indicated.

**Figure 3:**
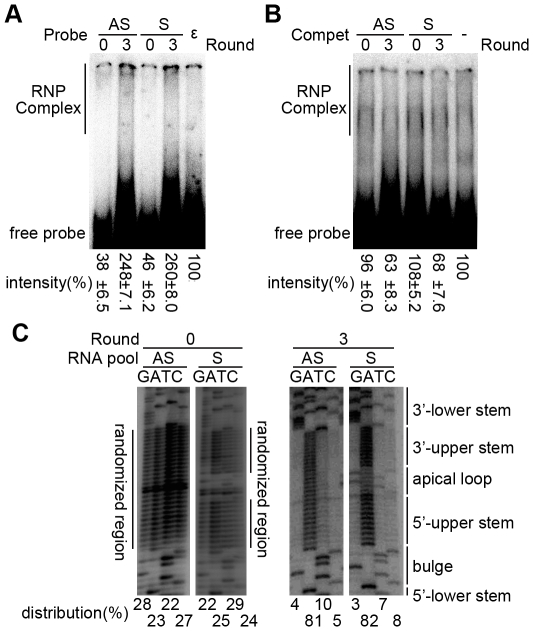
Enrichment of miniP binding aptamers after 3 selection rounds. (A). Increased miniP binding affinity. The unselected (0) and round 3 selected (3) RNA pools and wt ε RNA (ε) were 5′-^32^P-labeled and incubated at 50 nM concentration with miniP. Intensities of the upward shifted signals (marked by the lane labeled RNP complex) were determined by phosphorimaging and analyzed by OptiQuant software. The results are expressed as mean signal intensities (from three experiments) ± standard deviations relative to that of the control reaction with wt ε RNA which was set to 100%. (B) Increased miniP binding specificity. The indicated unlabeled RNA pools were added as competitors (Compet) in 20-fold molar excess over the labeled wt ε RNA probe. The signal from the uncompeted reaction was set to 100%. (C) Distribution of individual nt at the randomized positions in the starting pools and after 3 selection rounds. The RT-PCR products from the indicated RNA pools were directly sequenced. Relative nt distribution was calculated by determining, by phosphorimaging, the signal intensities in the randomized regions for each nt lane, and normalization to the signal intensities in the non-randomized region of the same lane. The results are expressed as percent relative to the sum of intensities over all 4 lanes which was set to 100%. Note the strong enrichment (>80%) of A residues at all randomized positions in the selected pools, and the preservation of the loop sequence in the S pool.

Chaperone-activated miniP protein was then used for three rounds (see below and Discussion) of *in vitro* aptamer selection from two RNA pools randomized at 23 (pool S) or the entire 29 positions in the upper stem (pool AS, see Fig. 2). In the S pool we maintained the 6 nt sequence encompassing the apical loop which is required for DHBV P *in vitro* priming activity but appears non-essential for HBV P-RNA binding [37]. Sequencing of the starting plasmid pools encoding the RNA libraries confirmed an approximately equal distribution of all four nt at the desired positions (Fig. 3C, left). The last step of an individual selection round is RT-PCR amplification of the P protein bound RNAs. As a precaution against artifactual selection of better amplifiable sequences as well as to promote amplification of the most enriched species in the selected pools RT-PCR amplification was restricted to the minimal number of cycles producing an easily detectable signal (12–18 cycles; see below).

Successful selection should result in the increasing enrichment of miniP binding individuals within the selected compared to the initial RNA pools. We therefore subjected the third round pools to EMSA, side-by-side with the starting pools and wild-type (wt) ε RNA as reference. To address binding affinities, all RNAs were radiolabeled and incubated with miniP plus chaperones. As shown in Fig. 3A, both the round 3 AS and S aptamer pools produced substantially more retarded signals than the parental pools. While an exact quantitation is inherently difficult given the broad signal distribution, comparison of the intensities by phosphorimaging (of the region labeled RNP complex in Fig. 3A) indicated an about 5- to 6- fold increase compared to the starting pools, which even exceeded the signals produced by the wt ε RNA by about 2.5-fold. This suggested the presence in the selected pools of aptamers with increased affinity for miniP. Increasing enrichment of miniP binding aptamers was further supported by the decreasing number of amplification cycles required to generate well detectable RT-PCR products at the end of each round, namely 18 cycles with the first round selected RNAs as template versus only 12 cycles with the third round RNAs. Furthermore, no amplification products were obtained from the SELEX control setup which contained everything except miniP. Hence the multiple chaperones present in the reaction did not by themselves contribute to RNA selection (data not shown).

To address binding specificity, we next used a competitive EMSA format in which the unlabeled pool RNAs compete with radiolabeled wt ε RNA for miniP binding (Fig. 3B), however only if they share the same binding site. Adding a 20-fold molar excess of the either the AS or S round 3 pool reduced the upward shifted signals to about 60–70% of the uncompeted wt ε RNA reaction whereas no reduction was seen with the unselected starting pools. These results revealed that miniP binding by the round 3 pool RNAs was specific.

In our previous SELEX experiments with DHBV P protein we had seen a rapid selection of C-rich consensus motifs in the upper stem already after 3 selection rounds which largely persisted through round 9 [29]. To examine the degree of enrichment in the current study, the nucleotide identities at the randomized positions in the round 3 pools were determined by direct sequencing. As shown in Fig 3C, a strong preference for adenine (A) was observed in either pool (with A representing >80% of the sequencing signal at all randomized positions), indicating that an open, rather than a base-paired, upper stem is beneficial for miniP binding. We therefore decided to isolate representative members of the round 3 pools and characterize them individually.

### Isolation of individual aptamers from round 3 RNAs pools

To obtain individual members from the round 3 pools, the RT-PCR products were cloned and 45 individual clones from about 500 were randomly picked and sequenced. Consistent with the pool sequence data, the vast majority (43 of 45) carried highly A-rich upper stem sequences (Table S1); the non-randomized apical loop sequence was maintained in the individuals from the S pool. Selection is evident from a direct comparison with the nt distribution in the unselected starting pools (Fig. 3C left). The remaining two clones carried additional insertions or deletions in the randomized part and were not further investigated.

**Figure 4:**
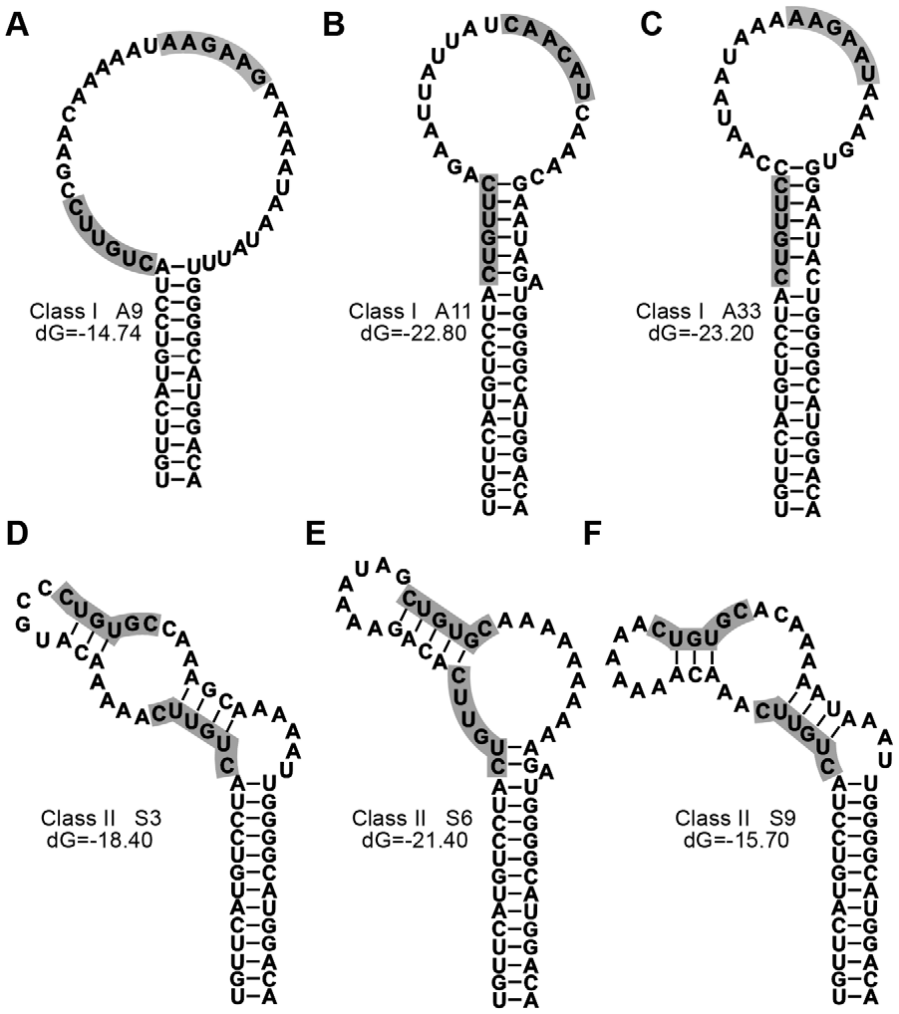
M-Fold analysis and representative predicted secondary structures of selected aptamer sequences. All selected aptamer sequences were analyzed using the M-Fold web server [39]. Default constraints were used which included a folding temperature of 37°C and an upper boundary of 50 on the number of computed foldings. (A–C). Lollipop-like class I structures of the indicated aptamers. The majority of pool AS-derived sequences belonged to this class. (D–F). Remotely ε-like class II structures of the indicated aptamers. Most members of this class were derived from pool S. For all structures, the calculated free energies are indicated (in kcal/Mol). A complete list of all sequenced individuals and their assignment to the different structure classes is provided in Table S1.

Because structural features in the ε RNA such as bulges and internal loops seem to be crucial for specific recognition by P protein [37], [38], all aptamer sequences were analyzed by the M-fold algorithm [39]. Based on common features of the predicted secondary structures the RNAs could be categorized into three classes. As shown in Table S1, 21 individual aptamers (∼49%) adopt a lollipop-like single stem-loop structure (class I) in which the former upper stem lacks any base-pairing (Fig. 4 A–C); the vast majority was derived from the AS pool with completely randomized upper stem. 20 aptamers (∼46%) adopt more complex, remotely ε-like structure with a lower stem, a bulge, and a partially base-paired upper stem (class II); most individuals derived from the S pool (with maintained apical loop sequence) belonged to this class (Table S1 and Fig. 4 D–F) The remaining 2 sequences (∼5%) lacked common secondary structural motifs and were classified as a separate group. Subsequently, three typical aptamers from class I (A9, A11, A33) and three from class II (S3, S6, S9) were chosen for further characterization.

### Exploration of potential decoy aptamers with high affinity and specificity for miniP

**Figure 5:**
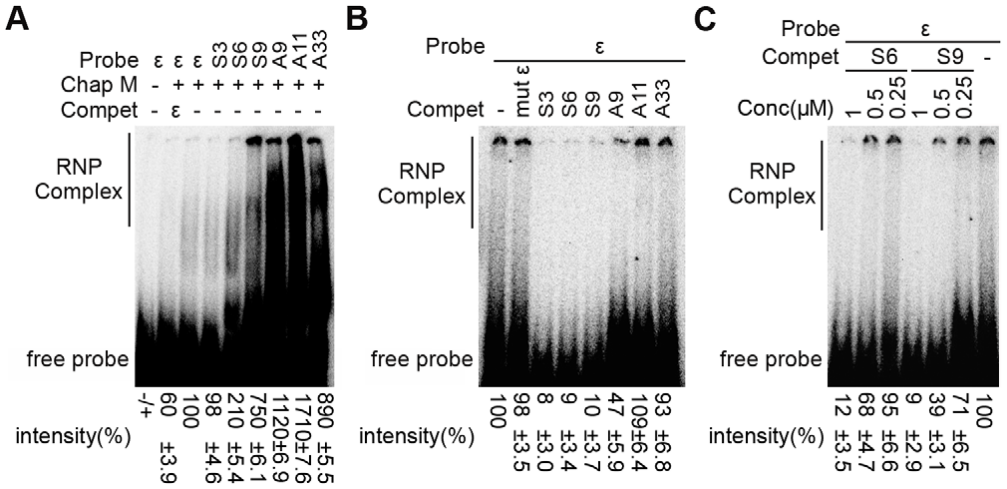
In vitro characterization of the interactions between miniP and individual aptamers. The indicated individual aptamers were tested for P protein binding affinity by direct EMSA (A) and for specificity by competitive EMSA (B and C) as described in Fig 3. A P protein binding-deficient mutant ε RNA (mut ε) served as negative control. (C) Dose dependence of EMSA competion by aptamers S6 and S9. EMSA competition was monitored as in Fig. 3B and 5B, except that the excess of competitor was successively reduced from 20 fold (1 µM competitor, 50 nM probe) to 10-fold and 5-fold. Relative signal intensities were determined as in Fig 3 and represent the mean from three experiments ± standard deviations.

The miniP binding affinities and specificities of the individual aptamers were assessed as for the pool RNAs by direct and by competitive EMSA. As shown in Fig 5A, aptamers A9, A11 and A33 led to intense shifted signals that nominally exceeded those produced by wild-type ε RNA by 9- to 17-fold, suggesting a strongly enhanced binding affinity. The class II variant S9 also produced strong shifted signals, whereas those generated by S3 and S6 were in the range of wild-type ε RNA (S3) or slightly higher (S6). Consistent with the previous pool RNA results, the data confirmed that individual RNAs isolated from the 3rd round pools are strong binders for miniP.

As competitors of wild-type ε RNA, however, the class II aptamers S3, S6 and S9 performed much better than the class I variants. The latter produced only modest (A9) or no signal reductions (A11, A33) whereas all three class II RNAs reduced the signals by about 90% at 20-fold molar excess (Fig. 5B); this decrease was even stronger than that seen with unlabeled wild-type ε RNA as competitor (about 40% reduction; Fig. 5A, lane 2). Expectedly, the non-binding mutant ε RNA had no effect. These data indicated that the class II aptamers, but less so or not at all the class I variants, bind to the same site on miniP as wt ε RNA, possibly due to the presence of the conserved apical loop sequence.

To corroborate binding specificity of the class II RNAs, we repeated the competition experiments with decreasing excess of the S6 and S9 RNAs (Fig. 5C). As before, the shifted signals generated by wt ε RNA were reduced by about 90% by either RNA at 20-fold molar excess; a 10-fold excess decreased the signals similarly as a 20-fold excess of unlabeled wt ε RNA (by 30 to 60%), and some reduction was seen for the S9 RNA even at only 5-fold excess. Given the difficulties in accurately quantitating the broadly distributed signals the latter reduction may not be significant; clearly, however, the data indicated a dose-dependent, specific inhibition of the formation of authentic wild-type ε RNA - miniP complexes.

### Analysis of the antiviral potential of aptamers

**Figure 6:**
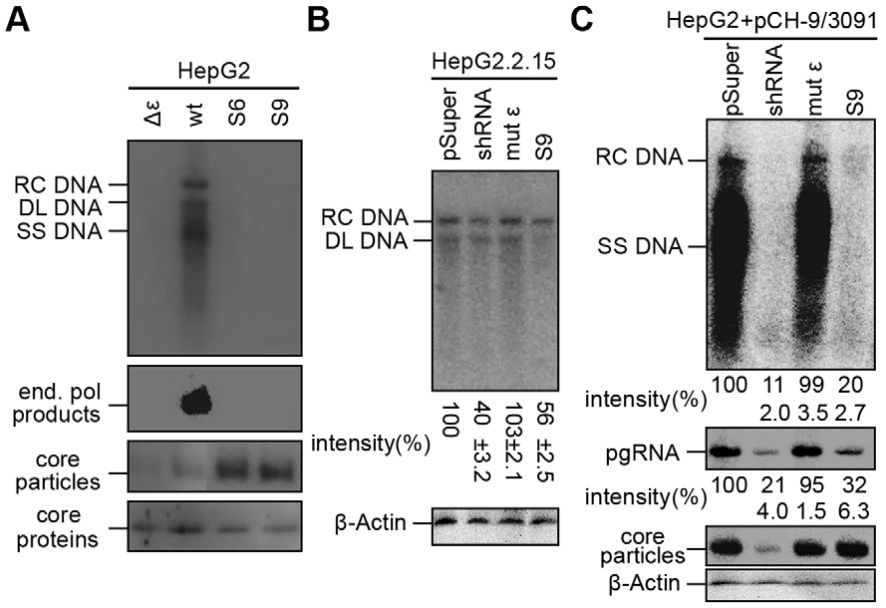
Functional analysis of potential decoy aptamers. (A) Aptamer sequences S6 and S9 do not support viral replication when in the context of a complete HBV genome. HepG2 cells were transfected with the wt HBV expression plasmid pCH-9/3091 (wt) or derivatives in which the authentic 5′ ε sequence had been deleted (Δε), or been replaced by the S6 and S9 sequences. Viral DNAs associated with cytoplasmic nucleocapsids were monitored by Southern blotting using a ^32^P labeled HBV DNA probe (top panel). The positions of relaxed circular (RC), double-stranded linear (DL) and single-stranded (SS) DNA are indicated. Capsids separated by native agarose gel electrophoresis (NAGE) were monitored by autoradiography after labeling via EPA (second panel) or by anti-capsid Western blotting (third panel). Core protein was monitored by Western blotting after SDS-PAGE (bottom panel). (B and C) Suppression of viral replication by an S9 aptamer expression vector. (B) Stably HBV producing HepG2.2.15 cells were transfected with pSUPER vectors encoding no RNA (pSUPER), an anti-HBV shRNA (shRNA), a P protein binding-deficient mutant ε RNA (mut ε), or the S9 aptamer RNA (S9). Viral replicative intermediates from cytoplasmic nucleocapsids were monitored by Southern blotting (top panel). The equal Western blot signals for β-actin (bottom panel) indicated that the lysates were derived from about equal numbers of cells. Note that the limited transfection efficiency of the pSUPER plasmids versus HBV production in all cells of the culture prevents a more pronounced suppression. (C) Strong suppression of viral DNA synthesis and pgRNA encapsidation despite maintained capsid levels in cells co-transfected with HBV and S9 expression vectors. HepG2 cells were cotransfected at a 1∶1 molar ratio with pCH-9/3091 and the indicated pSUPER vectors. Effects on viral DNA synthesis were monitored by Southern blotting (top panel). Encapsidated viral RNA was monitored by Northern blotting (second panel). Cytoplasmic capsids were detected by Western blotting after separation by NAGE (third panel). β-Actin levels in the cytoplasmic lysates from which the encapsidated viral RNA and capsid samples were derived were monitored by Western blotting after SDS-PAGE.

The *in vitro* data described above suggested that in particular RNA variants S6 and S9 might be suitable as decoys to compete for P protein binding with the authentic ε signal present on the pgRNA. To explore this possibility, we first tested whether the variant sequences were able to support viral replication in the context of a complete HBV genome. To this end, we replaced the 5′ proximal ε sequence in the wt HBV encoding vector pCH-9/3091 [40] by the S6 and S9 sequences. An analogous plasmid lacking 5′ ε (Δε-HBV) served as negative control**.** The amino acid exchanges in the core protein (D4E, P5A) caused by introduction of a Hind III restriction site did not detectably affect capsid assembly (Figure. 6A, panel labeled “core particles”). Following transient transfection into HepG2 hepatoma cells, synthesis of viral DNA within newly formed cytoplasmic core particles was examined by Southern blotting. The wt HBV plasmid generated the expected replicative intermediates, i.e. relaxed circular DNA (RC-DNA), double-stranded linear DNA (DL-DNA) and single-stranded DNA (SS-DNA) plus probably incompletely extended double-stranded products (Fig. 6A, top panel). Expectedly, no signals were observed with the Δε-HBV construct, and neither from the constructs carrying the S6 and S9 sequences. This was not caused by a lack of core protein production or assembly, since comparable Western blot signals were seen in lysates from all four transfections (Fig. 6A, lower two panels). Failure of the variant sequences to support replication was independently confirmed by an endogenous polymerase assay (EPA) in which initiated DNA strands are extended by the encapsidated P protein upon provision of exogenously added dNTPs. A specific signal was exclusively produced from the wt HBV construct (Fig. 6A, second panel).

Finally, as a proof-of-principle for the desired applicability of the SELEX-derived aptamers we generated a pSUPER-derived [41] RNA polymerase III H1 promoter vector to express the S9 RNA in cells. An analogous vector encoding the non-P binding mutant ε RNA served as negative control, and a pSUPER vector encoding an anti-HBV shRNA directed against the DR1 region (1826–1845nt) of the HBV genome, previously shown to potently down-regulate HBV replication [42], as positive control.

First, we transfected the three RNA vectors and, as a further control, the empty pSUPER plasmid into the stably HBV producing HepG2.2.15 cell line and monitored viral DNA synthesis by Southern blotting (Fig. 6B). Quantitation by phosphorimaging revealed, compared to the wt ε RNA and empty vector controls, a reproducible about 40–50% inhibition by the S9 RNA which was similar to that achieved by the shRNA (about 60%). The about equal signals for β-actin in all lysates indicated that the reductions were not due to major cytotoxic effects. Importantly, this experimental set-up underestimates the potency of the S9 RNA (and the shRNA) because all cells produce virus whereas the inhibitor is made only in the fraction of transfected cells (which for the HepG2.2.15 cells was routinely around 20–30%, as estimated from the number of GFP positive cells upon transfection with an eGFP expression vector; data not shown). We therefore repeated the experiments in a cotransfection setting whereby both the target HBV plasmid and the RNA inhibitor plasmid are delivered into the same fraction of cells.

To this end, plasmid pCH-9/3091 and the pSUPER vectors were cotransfected at a 1∶1 ratio into naive HepG2 cells and viral replication was assessed by Southern blotting (Fig. 6C, top panel). Quantitation by phosphorimaging revealed similarly high levels of replication in the cells that had receivied the wt ε RNA vector and the empty pSUPER plasmid; in contrast, the S9 RNA vector caused a reduction of replicative intermediates by about 80–85%, similar to that achieved by the shRNA vector (∼90%). Comparable inhibitions were seen in independent repeat experiments. Analyzing the amounts of encapsidated viral RNA gave similar, though slightly less pronounced results (Fig. 6C, second panel). The mutant ε RNA vector had virtually no effect compared to the empty pSUPER plasmid whereas both the S9 RNA and the shRNA vector strongly reduced the signals. Notably, for the shRNA vector, this correlated with a strong reduction in capsid levels (Fig. 6C, third panel). This was expected because the shRNA targets all viral transcripts including the pgRNA which also serves as mRNA for the core protein. In contrast, capsid signals were not significantly weakened by the S9 RNA vector. This is fully consistent with the S9 aptamer competing with the authentic ε signal on the pgRNA for P protein binding. Hence less pgRNA is encapsidated, and this results in decreased levels of encapsidated viral DNA. Lastly, the comparable amounts of β-actin present in the cytoplasmic lysates (Fig. 6C, bottom panel) from which the capsid samples and capsid-borne pgRNA samples were derived confirmed the presence of similar numbers of cells in all four experiments. Together with the unaffected capsid levels in the S9 aptamer-treated cells this made it unlikely that major cytotoxic effects were responsible for the selective reduction in encapsidated viral RNA and DNA.

To directly address potential cytotoxicity of the aptamer RNA, we compared cell viability in non-transfected HepG2.2.15 and HepG2 cells versus the same cells after transfection with the different pSUPER plasmids (HepG2.2.15) or co-transfection with pCH-9/3091 plus the pSUPER plasmids (HepG2). Using a commercial MTT assay, no significant differences were seen; the original data from three independent determinations are shown in Table S2. Hence the strong suppression of pgRNA encapsidation and DNA synthesis by the S9 aptamer RNA is not due to unspecific cytotoxic effects.

## Discussion

The multifunctional interaction between HBV P protein and the ε signal is central for viral replication. Based on a newly established P-ε reconstitution system, we report here for the first time the *in vitro* SELEX-based screening for and characterization of aptamers which specifically suppress the P-ε interaction in human HBV.

Our choice of using, in addition to the AS pool with completely randomized upper stem (AS), the more constrained S pool with preserved apical loop sequence (S) was intended to account for as yet poorly understood differences in P-ε complex formation between DHBV and HBV. Regarding virus replication in cells, mutational studies suggest that the loop is essential for pgRNA encapsidation and initiation of reverse transcription in both viruses [11], [43], [44]. For DHBV, this also holds *in vitro* for ε RNA binding and ε dependent priming [38]. For HBV, in contrast, *in vitro* binding to P protein does not require the loop, as indicated by upward shifted EMSA signals by numerous ε variants, including one with a complete deletion of the loop [37].

A plausible explanation is that formation of a priming-active P-ε complex is a dynamic, sequential process in which an initial, loop-independent binding event, likely mediated by the bulge region [37], [45], is followed by a rearrangement in the RNA during which the loop makes important new contacts to P protein. For DHBV, this rearrangement can occur *in vitro* whereas the biophysically proven [3], [46] much more rigid and stable structure of the upper stem in wt HBV ε prevents the rearrangement, halting RNP formation at the first step. Consistent with this view, we have shown that the DHBV ε RNA adopts a new, more open upper stem structure in priming-competent RNPs [17], [47]. Furthermore, several of the DHBV ε variants with low base-pairing potential in the upper stem from our previous SELEX study were active *in vitro* priming [29] and even *in vivo* [30] whereas artificial stabilization of the upper stem abrogated priming activity [29], [38]. Two data sets from our current study further support such an interpretation. First, the enrichment of A residues seen with both starting pools suggests a counterselection against stable upper stem structures, analogous to the previous results with DHBV [29]. Second, although the non-constrained AS pool members A9, A11 and A33 showed very strong binding to miniP (Fig. 5B), the S pool-derived aptamers S3, S6 and S9 with preserved loop sequence were all three much better competitors (Fig. 5B and 5C). This suggests that also for HBV the loop contributes to specific P protein binding. This retrospectively justifies inclusion of the constrained S pool in the current study and is an important consideration for development of further improved ε decoys.

Regarding miniP as capturing agent for P binding RNAs, our initial data indicated that detectable ε binding required chaperones (Fig. 1), in accord with previously published data [31], [37] yet in apparent contrast to the absence of such a chaperone-dependence with similarly truncated DHBV miniP [32], [33], [48]. This may either relate to differences between the two P proteins, or reflect a general rather than a specific [35], [49] chaperoning effect in the HBV system. Regardless of the exact mechanism, the multicomponent nature of our SELEX system could have led to the selection of chaperone-binding RNAs. However, we never observed detectable RT-PCR products in control reactions containing all components except miniP, a first hint that selection was specific for P protein.

Specificity was further confirmed by the successive increase in miniP binding and ability to compete with wt ε RNA from the non-selected to the round 3 selected RNA pools (Fig. 3A, 3B), the evident enrichment of A-rich rather than random upper stem sequences (Fig. 3C), and the miniP binding competence of individual round 3 sequences derived from both the AS and S pools (Fig. 5A). Probably the most convincing evidence that the *in vitro* miniP SELEX system mimics authentic features of the P-ε interaction is the strong inhibition of viral replication by S9 RNA (see below) which *in vitro* combined high affinity with high specificity of miniP binding.

Currently we do not know whether S9 represents the optimal combination of these properties. A frequently used strategy to find ever better binding aptamers, and sometimes a single winning sequence, is to increase the number of selection cycles [23], [29], [50]. In our previous DHBV SELEX study we followed this approach over 9 rounds. However, after rapid selection of a small pool of DHBV P protein binding ε RNAs with open upper stems during the first 3 rounds, little further reduction in pool complexity was observed in the subsequent rounds (see Suppl. Fig. 5 in [29]); rather, several sequences with comparable P binding properties coexisted. While this does not exclude that additional selection rounds in the current study would have yielded even more potent aptamers than S9, we consider selective screening of the already isolated class II aptamers for antiviral activity as a more promising alternative. Direct structural analyses may then also reveal which particular sequence and/or structure features correlate with the highest affinity and specificity for P protein.

Most importantly, the P binding properties of the S9 aptamer implied by the *in vitro* results, namely higher affinity than wt ε RNA (Fig. 5A) and more efficient competition with labeled wt ε RNA (Fig. 5C), translated into a significant inhibition of viral replication by the S9 RNA in transfected cells (Fig. 6), providing proof-of-principle for the feasibility of the HBV e decoy approach.

Cotransfection of the S9 RNA vector with the HBV expression plasmid caused an 80–85% reduction in viral replicative DNA intermediates, comparable to that achieved by a potent anti-HBV shRNA vector (∼90%) but by a different mechanism. The shRNA vector simultaneously reduced capsid levels whereas the S9 vector did not. Although we have not directly determined total viral transcript levels, maintainance of similar capsid levels in the S9 treated versus the control cells suggests that similar levels of pgRNA as mRNA for core protein were available. However, the amounts of pgRNA per capsid were strongly reduced (Fig. 6C), as expected if the S9 RNA competed for P protein with the authentic ε signal on pgRNA. The shRNA, by contrast, directly targets the viral transcripts [42]. Because reverse transcription of the pgRNA occurs inside capsids, it is only consequent that fewer pgRNA containing capsids in the S9 treated cells also produce less encapsidated viral DNA. Notably, inhibition of HBV replication by the S9 aptamer was not restricted to transiently transfected cells, but was also detectable in the stably HBV producing HepG2.2.15 cell line (Fig. 6B). The less pronounced antiviral efficacy is in line with the fact that only a fraction of the HBV producing cells receive the antiviral RNA; hence stronger effects than those observed would not have been expected.

A peculiar feature of hepadnaviruses is the cis-preferential packaging of the same pgRNA molecule that served to translate P protein [16]; this preference is not absolute because pgRNAs defective for P protein production can be packaged if P protein is provided in trans from a separate mRNA. However, it provides an extra hurdle that any ε decoy has to leap. Inhibition of viral replication by the S9 aptamer vector but not the mutant ε RNA vector suggests that the high affinity of the S9 RNA is one important factor. Another is the intracellular concentration of the pSUPER expressed RNAs which, despite use of the same polymerase III promoter, might be influenced by different synthesis and/or degradation or processing (e.g. by DICER-like activities) rates. For any potential therapeutic application, these parameters will have to be addressed in detail.

This holds as well for other application-relevant issues, including appropriate *in vivo* delivery systems, and potential adverse effects of the S9 RNA and alike inhibitors. Although we saw no signs of cytotoxicity in S9 RNA vector transfected cells (Fig. 6C, and Table S2), this may be different in a live organism; we therefore plan to test the antiviral potency of the S9 RNA and potential further improved ε decoy aptamers in HBV transgenic or hydrodynamically HBV transfected mice. Notably, a TAR RNA decoy aptamer is part of ongoing clinical ex vivo gene therapy studies against HIV-1 infection [51], indicating that these technical challenges are surmountable.

In conclusion, our study provides proof-of-principle for the feasibility of an ε decoy approach as a novel strategy to combat chronic hepatis B. While various improvements will be required for therapeutic application, the large number of people suffering from this disease, the obvious limitations of current therapies and the fact that P-ε complex formation represents a completely different target for intervention make such efforts highly worthwhile.

## Materials and Methods

### Bacterial strains and plasmid constructs

*E. coli* strains, DH5a and BL21-CodonPlus(DE3), were used as the host strains to clone and express HBV miniP protein, respectively.

The parental vector used to construct complete HBV genomes carrying aptamer sequences was pCH-9/3091, which contains a slightly overlength HBV genome under control of the CMV promoter [14]. As a recipient for the different aptamer sequences, we first generated plasmid pCH-9/3091Δ which carries a deletion in the 5′-ε signal sequence. In brief, an ∼1.4-kb restricted PCR amplified Hind III-Xho I fragment (nt 11-1409) and an ∼2.4-kb restricted PCR amplified Hind III- Sca I fragment (nt 3918-6281) acquired from the corresponding regions of pCH-9/3091 were simultaneously cloned to the ∼2.5-kb restricted Xho I-Sca I backbone fragment (nt 1410-3917) to produce the pCH-9/3091Δ vector. The Hind III site introduced by the PCR primer replaced the dinucleotide CC (positions 1912 and 1913 of the HBV sequence, NC_003977.1) by AG. In addition, we created a unique EcoR V restriction site between DR1 and 5′- ε by replacing the CTA residues (positions 1835 to 1837) within a unique primer with GAT (gatATC, mutated positions in lowercase). The respective pCH-9/3091 vectors were then constructed by inserting PCR amplified EcoR V - Hind III restricted aptamer fragments into pCH-9/3091Δ restricted with the same enzymes.

The resulting plasmid pCH-9/3091B was then used to construct pCH-9/3091-aptamer vectors by inserting the restricted PCR amplified Eco RV-Hind III aptamer fragments. Plasmid pCH-9/3091Δε was made by replacing the ∼1.4-kb Hind III-Xho I fragment (nt 11-1409) within pCH-9/3091Δ for a restricted PCR amplified Hind III-Xho I fragment (nt 1-1409), which carries the start codon of translation of core protein and the identical displacement of CC for AG. RNA expression vectors were constructed by transferring the restricted PCR amplified Hind III-Xho I aptamer or ε binding-deficient mutant (shown in Fig. 2) [37] fragments into pSuper. All constructs were confirmed by sequencing the relevant region on the plasmids.

### Expression and purification of the miniP protein

The miniP protein was expressed from pET-MBP-TEV-HP1-199/292-601 in BL21-CodonPlus(DE3) as His-tagged fusion protein with the maltose-binding protein (MBP, at the N-terminus) and purified as previously described [33].

### In vitro transcription

T7 RNA polymerase mediated run-off transcription was performed as described previously [38]. The starting AS and S variant RNA pools (Fig. 2) were generated by annealing the (−)-polarity oligonucleotides DepsNuppAS(−) (5′-GGTACCTGTCCATGCCCCA(N)_29_GAACAGTAGGACATGAACAGCCCTATAGTGAGTCGTATTAattc-3′) and DepsNuppS(−) (5′-GGTACCTGTCCATGCCCCA(N)_12_GCACAG(N)_11_GAACAGTAGGACATGAACAGCCCTATAGTGAGTCGTATTAattc-3′), respectively with a (+)-polarity T7 promoter oligo; randomized positions are indicated by underlined N. The resulting partial duplex DNAs were used as templates for *in vitro* transcription using the T7 MEGAshortscript kit (Ambion). The mutant RNA, which has been previously proved to be an *in vitro* P binding-deficient RNA [37], was similarly synthesized by using the (−)-polarity DepsNuppε-mut(−) (5′-GCCCCAAAGCCACCCAAGGCACAGCTTGGAGGCTTGAcagaTAGGACCCCTATAGTGAGTCGTATTAattc-3′) as transcription template; the mutated positions are indicated by underlined lowercase. The wt HBV ε RNA was obtained by *in vitro* transcription of the corresponding plasmid (pBS-A1) after linearization with Eco RI of the above described sequences. The products were analyzed by electrophoresis in 12% denaturing polyacrylamide gels, followed by silver staining; RNA concentrations were determined by measuring the absorbance at 260 nm. Subsequent RNA pools were produced analogously, but using the duplex RT–PCR products as template.

### In vitro reconstitution of the miniP-RNA complexes

In a 30 µl *in vitro* binding reaction for selection of strong miniP-binders, approximately 100 ng purified miniP proteins were incubated with Hsp90β (1 µg, Abcam), Hsp70 (10 µg, Biovision), Hdj1 (0.6 µg, Biovision), Hop (1.2 µg, Biovision), and p23 (0.3 µg, Abcam), together with the randomized RNAs at a final concentration of 12286M. For a 102286l *in vitro* binding setup for EMSAs-based detection, the amount of individual proteins was exactly added as described recently [31]. For the P protein negative control, the miniP protein was omitted. The reactions were incubated for 2 h at 30°C to allow for formation of miniP-RNA complex.

### Isolation of miniP-binding RNAs

Isolation of miniP-binders was performed as previously described [29]. Briefly, 400 µl binding buffer (0.1 M sodium phosphate, pH 7.4, 150 mM NaCl, 20 mM imidazol, 0.1% (v/v) NP-40, 100 mg/ml yeast tRNA) containing 50 µl Ni^2+^NTA agarose beads (Qiagen) were added to the *in vitro* reconstitution reactions [17], and incubated for one more hour. To remove unbound and weakly bound RNAs and chaperone components, the beads were washed twice with 1 ml each of ice cold binding buffer, then twice with 1 ml each of TMK buffer (50 mM Tris/HCl, pH7.5, 10 mM MgCl_2_, 40 mM KCl, 100 mg/ml yeast tRNA). Finally the beads were suspended in 100 µl TMK buffer and the bound RNAs were purified by phenol extraction. The extracted RNAs were precipitated, and dissolved in 15 µl TE buffer.

### RT–PCR and direct sequencing

An aliquot of 2 µl of the isolated RNA solution was reverse transcribed using the reverse primer (5′-CAATCTGCAGTCTAGATAAGGTACCTGTCCATGCCCCA-3′) and M-MLV reverse transcriptase (Promega) as per the manufacturer's instructions. Subsequently, the RNA template was degraded by alkaline hydrolysis and an aliquot of this solution was amplified using Taq DNA polymerase (Promega). RT–PCR products were directly sequenced using the Thermo Sequenase Cycle Sequencing Kit (USB, Cleveland, OH) as recommended by the supplier. The RT-PCR products from the third selection round were cloned into pUC19 vector (Invitrogen) via the terminal Xba I and Eco RI sites and plasmid DNAs from 45 individual colonies were sequenced.

### Radioactive labelling of RNA and EMSAs

RNAs were 5′ terminally labeled by dephosphorylation and rephosphorylation with γ-^32^P ATP (3000 Ci/mmol) as described [38]; free γ-^32^P ATP was removed using Quick Spin columns (Roche). For direct EMSA (protein-binding affinity), the ^32^P-labelled RNAs were used at 50 nM final concentration (specific activity ∼2×10^5^ cpm/pmol). For competitive EMSA (protein-binding specificity), a mixture of ^32^P-labeled wt ε RNA (50 nM final concentration) plus 1 µM unlabelled selected RNAs was used. Following incubation, the samples were analysed on 5% (w/v) polyacrylamide (37.5∶1 acylamide:bis acrylamide) gels containing 0.5× TBE. Labeled RNAs and RNP complexes were detected by autoradiography of the dried gels. Signal intensities were determined by phosphorimaging using OptiQuant 5.0 software (Perkin Elmer).

Relative quantitative analysis was performed to calculate intensities of the shifted signals using OptiQuant 5.0 (PerkinElmer).

### Cells, transfections and isolation of core particles

HepG2 [52] and HepG2.2.15 [53] cells were maintained in Dulbecco's modified eagle's medium supplemented with penicillin and streptomycin, and 10% fetal bovine serum. Transfections with pCH-9/3091 and its derived constructs were performed using Lipofectamine^TM^ 2000 (Invitrogen) according to the manufacturer's instructions, using 24 µg DNA per 10 cm diameter plate. pSUPER constructs were analogously transfected using 8 µg DNA per 6 cm diameter. For co-transfections, mixtures of 12 µg pCH-9/3091 and 12 µg pSUPER plasmid per 10 cm diameter dish were used. Cytoplasmic core particles were isolated from transfected-cells as previously described [54], with minor modifications. Briefly, 48 h after transfection, cells were lysed in either 1 ml (10 cm dish) or 600 µl (6 cm dish) Nonidet P-40 lysis buffer (10 mM Tris–HCl [pH 8.0], 50 mM NaCl, 1 mM EDTA, 1% Nonidet P-40). The clarified lysates were adjusted to 10 mM final concentration of MgCl_2_, and incubated with 20 U DNase I (Fermentas) plus 15U RNase A (Fermentas) at 37°C for at least 6 h. Cytoplasmic core particles were then precipitated with 6.5% polyethylene glycol.

### Southern blotting and endogenous polymerase assay (EPA)

To analyze HBV DNAs by Southern blotting, the isolated core particles were incubated with 50 U micrococcal Nuclease S7 (Fermentas) to remove the nonencapsidated DNA completely. Then core DNA was extracted, separated by 1% agarose gel electrophoresis, and hybridized to a ^32^P-labelled random-primed probe specific for the HBV sequence.

EPA was performed as previously described in [54] with minor modifications. In brief, isolated core particles were incubated at 37°C at least 3 h with EPA reaction buffer (50 mM Tris–HCl [pH 7.5], 75 mM NH_4_Cl, 1 mM EDTA, 25 mM MgCl_2_, 0.1% h-mercaptoethanol, 0.5% Nonidet P-40) supplemented with 0.5 mM each of dCTP, dGTP, and dTTP, and 10 µCi α-^32^P-dATP (3000 Ci/mmol). The resulting ^32^P-labeled reaction mixtures were directly electrophoresed on a 1% native agarose gel and then subjected to dry gel autoradiography.

### Western blotting

For native western blotting, isolated core particles were electrophoresed on 1% TAE agarose gels and transferred to PVDF membrane. To normalize the transfection efficiencies, aliquots corresponding to 20 µl of 1 ml cytoplasmic lysate from a 10 cm diameter plate were subjected to electrophoresis in 15% SDS polyacrylamide gel and transferred to PVDF membrane. Immunoblotting was performed using antibodies against native and denatured HBc (both from DAKO). Horseradish peroxidase-conjugated anti-rabbit secondary antibody and enhanced chemical luminescence (ECL) were employed to visualize either assembled HBV core particles or translated core proteins. For normalization of the HBV signals to the number of cells, the housekeeping protein β-actin present on the same blots was detected using an anti-β actin antibody (Abcam).

### Northern blotting

Viral pgRNA from intracellular core particles was prepared as described in [55], and analysed by Northern blotting using random-primed ^32^P DNA probes specific for HBV.

### Cell viability assay

24 h post-transfection, aliquots corresponding to 1/10^th^ transfected cells from a 10 cm-diameter plate were suspended in 1 ml DMEM medium, of which 100 µL were seeded into the wells of a 96-well plate. MTT assays were performed using the Vybrant® MTT Cell Proliferation Assay Kit (Invitrogen) according to the manufacturer's instructions.

## Supporting Information

Table S1 **Classification of individual isolated RNA aptamers.**
(DOC)

Table S2 **Transfection of pSUPER vector encoding the S9 aptamer is not detectably cytotoxic.**
(DOC)
