# Supplementary material for: A SELEX-Screened Aptamer of Human Hepatitis B Virus RNA Encapsidation Signal Suppresses Viral Replication
Source: PLoS One. 2011 Nov 18;6(11):e27862. doi: 10.1371/journal.pone.0027862 (PMC3220704; doi:10.1371/journal.pone.0027862)
Supplement: Table S1 — Classification of individual isolated RNA aptamers. (DOC) [file pone.0027862.s001.doc]

**Supplementary Table 1:** Classification of individual isolated RNA aptamers

| **Groups** | **aptamer** | **Random sequences** | **proportion** |
| --- | --- | --- | --- |
|  | wt ε RNA | AAGCCUCCAAGCUGUGCCUUGGGUGGCUU | Clones (%) |
| I | A1 | AUAAAUAAGUAAUACACAAUAAAAAAUAU | 21 (49%) |
|  | A2 | CAAAAAAAAAAAAAAAAAACAGAACAAUG |  |
|  | A3 | CACAACGAAACAAUAACAAUAAUAACAAG |  |
|  | A5 | AAAUAAGAAGAAUAAAAACAAAAAGACAA |  |
|  | A7 | AAUAAAAAUAAUAAUAAAAAAAAAAUAGA |  |
|  | A8 | AAAUAACAAUAAUAAAAAACCAAAAACAA |  |
|  | A9 | CGAACAAAAAUAAGAAGAAAAAUAAUAUU |  |
|  | A10 | AAAAUAACAAUAAUAAUAAAAAAAAACAA |  |
|  | A11 | AGAAUUAUUAUCAACAUCAAACGAAUAGA |  |
|  | A12 | AAGAAAAAAAAAAAAAAAAAAUAAAAAUA |  |
|  | A13 | AUGAUAAAAAAAAUAAUAAAUAUGAAUCA |  |
|  | A16 | AGAAAAAACAAUAACAAUAAAACAACAAC |  |
|  | A19 | AAAAAACAACAAUAAUAAAAAAAAACUUA |  |
|  | A20 | CGGAUAAAACCAAUAACAAAAUGAAACGA |  |
|  | A22 | CAAAGAAUAAUAAUAAUAAAAGGAACAAA |  |
|  | A23 | AAAAAACAAUAAUAACAAAAAAAAAAACA |  |
|  | A28 | AAUAAUAAAAAGAAUAAAGAAACAACAAA |  |
|  | A31 | ACAAAACAAUAAAAACAAAAAAAAAUAAC |  |
|  | A32 | ACAUAUAAAAACAACAAAAAACAAAAUAU |  |
|  | A33 | CCAAUAAUAAAAAGAAUAAAGUGGAAUAC |  |
|  | S7 | AAAAUAUAAAACUGUGCAAAAAAAAUCAA |  |
| II | A4 | AAAAUAAUUAAUAAUAAUAAAAUAAUUAU | 20 (46%) |
|  | A6 | AAUAACAAUAAAAACAAAAAAAACAGGUA |  |
|  | A14 | AGCUCUAUCACAACUCAAGAACAGAUCUA |  |
|  | A15 | AUGGCAAGCCAACAUAAAAAAAAAUAAUA |  |
|  | A17 | AAAAAAAAAAACAAUAAUAAACAUUGAAA |  |
|  | A18 | AAUAACAACAAUAACAAAGAACAUGCAAA |  |
|  | A21 | AAUAACAAAAACUAUAAGAGAAACAAAAA |  |
|  | A24 | AAUAAAAACAACAAAAUCAAACAACAUAA |  |
|  | A25 | AUAAAAACAAUAAGAAUACCAAGAAAGAA |  |
|  | A26 | AGUAUAUAACAAUAAUAAUA-GAAUAAAU |  |
|  | A27 | UUGAUAACAAAAGCAAAAACUAGUUCACA |  |
|  | A29 | CAAGCCUACAAAAACAAUAAAAAAAGUAC |  |
|  | A30 | AAAAAUAAUAAAAACAAAAAAGUAUAAAA |  |
|  | S1 | AGCAAAAAAGACUGUGCAAAACAACAAUA |  |
|  | S2 | AAUAUUAAAGACUGUGCAAAAAAAAAAAA |  |
|  | S3 | AAAAACAUGCCCUGUGCCAAAGCAAAAAU |  |
|  | S5 | AACAUAUCUAACUGUGCAUAAAAUAAUCA |  |
|  | S6 | ACAGAAAAUAGCUGUGCAAAAAAAAAAGA |  |
|  | S8 | AGAAAAAAACACUGUGCAAAAGAAAAAAA |  |
|  | S9 | AAACAAAAAAACUGUGCACAAAAAUAAAU |  |
| III | S4 | AAAACAAACAGCUGUGCAAAAAUAAAAAA | 2 (5%) |
|  | S10 | AAACACACAAACUGUGCACAAGCAUCAGA |  |

Of the 43 aptamer sequences, 33 (A1-33) were isolated from the AS pool, 10 (S1-10) were from the S pool. Only the randomized region (5'–3') is indicated, with the nt of the apical loop given in underlined letter. The proportion of isolated clones is specified, and values are given as n (%).
