# Supplementary material for: A SELEX-Screened Aptamer of Human Hepatitis B Virus RNA Encapsidation Signal Suppresses Viral Replication
Source: PLoS One. 2011 Nov 18;6(11):e27862. doi: 10.1371/journal.pone.0027862 (PMC3220704; doi:10.1371/journal.pone.0027862)
Supplement: Table S2 — Transfection of pSUPER vector encoding the S9 aptamer is not detectably cytotoxic. (DOC) [file pone.0027862.s002.doc]

**Supplementary Table 2: Transfection of pSUPER vector encoding the S9 aptamer is not detectably cytotoxic.**

| **Cell line** | **Lipofectamine^TM^ 2000** | **Plasmid(s)** | **Absorbance** |
| --- | --- | --- | --- |
| HepG2.2.15 | + | - | 0.717±0.005 |
|  | + | pSUPER | 0.708±0.008 |
|  | + | shRNA | 0.691±0.004 |
|  | + | mut ε | 0.686±0.002 |
|  | + | S9 | 0.685±0.005 |
| HepG2 | + | - | 0.714±0.007 |
|  | + | pCH-9/3091+ pSUPER | 0.691±0.003 |
|  | + | pCH-9/3091+ shRNA | 0.666±0.006 |
|  | + | pCH-9/3091+ mut ε | 0.652±0.004 |
|  | + | pCH-9/3091+ S9 | 0.650±0.003 |

Cells were transfected with the indicated pSUPER vectors (HepG2.2.15) or cotransfected with pCH-9/3091 and the pSUPER vectors (HepG2), and cell viability compared to the untreated control cells was assessed 24 h post transfection using a commercial MTT assay. The results are given as the mean absorbance at 490 nm ± standard deviation from 3 independent determinations. No significant differences were detectable.
